# Parasitic mixing in photomixers as continuous wave terahertz sources

**DOI:** 10.1038/s41598-024-55661-x

**Published:** 2024-03-06

**Authors:** Michael Kocybik, Maris Bauer, Fabian Friederich

**Affiliations:** 1 Department of Materials Characterization and Testing, Fraunhofer Institute for Industrial Mathematics ITWM, 67663 Kaiserslautern, Germany; 2 Department of Physics and Research Center OPTIMAS, RPTU Kaiserslautern-Landau, 67663 Kaiserslautern, Germany

**Keywords:** Optical techniques, Engineering

## Abstract

We present observations of parasitic frequency components in the emission spectrum of typical photomixer sources for continuous wave (CW) terahertz generation. Broadband tunable photomixer systems are often used in combination with direct power detectors, e.g., for source and/or detector characterization. Here, spectral components besides the intended terahertz emission at the difference frequency of the two excitation lasers can significantly distort the measurement results. In this work, the appearance of parasitic mixing signals is observed in broadband measurements with a broadband antenna-coupled field-effect transistor as terahertz detector (TeraFET). The measurements reveal weaker spectral absorption features than expected and also a signal plateau towards higher frequencies, both strongly indicating a background in the detection signals. The photomixer emission is investigated in detail with a terahertz Fourier-transform infrared spectrometer (FTIR). We relate the observed parasitic frequency components with good quantitative agreement with the mode spectra of the semiconductor lasers. We also present one possible approach to overcome some of the issues, and we emphasize the importance of our findings to avoid distorted measurement results. To our knowledge, the essential aspect of parasitic mixing has so far been largely ignored in the literature where terahertz CW photomixer emitters are widely used for spectrally resolved measurements.

## Introduction

Terahertz Technologies have proven suitable for many applications in the fields of, e.g., non-destructive testing, wireless communication, and spectroscopy^1,2^. This success is to a large extent based on ongoing research and development of technological devices and systems for this type of electromagnetic radiation, both on the side of terahertz generation as well as detection. In order to put newly developed components into use, a proper characterization of device properties is crucial. On the side of radiation sources, such parameters can be, e.g., the total output power, the frequency bandwidth for broadband systems, pulse lengths, the linewidth of continuous wave (CW) sources, tuning ranges, and others. On the detection side, one of the most important figures of merit is usually the (spectrally resolved) sensitivity expressed, e.g., as noise-equivalent power (NEP). In detail, the NEP is the detectors noise spectral density in a one hertz bandwidth—deduced from noise measurements or theoretical considerations^3^—divided by the detector’s output response to a given incident radiation power, i.e., it’s responsivity expressed as amperes or volts per watt. It becomes apparent that for a precise characterization of terahertz detectors, the knowledge of, in particular, the frequency characteristics of an employed terahertz emitter are essential. Furthermore, the current intensive development of 6G technologies places extreme demands on solutions in the field of high-frequency measurement technology, in which photonic concepts are becoming increasingly relevant and require a far more comprehensive characterization of the measurement system^4,5^. At the same time, CW terahertz photomixing is also gaining increasing relevance in the field of non-destructive testing^6,7^ and requires very detailed knowledge of the system properties, for example, for use in the field of layer thickness measurement in order to obtain precise results^8,9^.

In many of todays experiments for detector characterization, photomixer devices are used as frequency-tunable terahertz CW sources for spectrally resolved measurements of the NEP of the device under test^10–15^. Their working principle is the generation of modulated charge currents by laser-excitation of semiconductor structures, usually photoconductors or photodiodes^16–21^. For CW terahertz generation, the output of two wavelength-tunable, single-mode optical lasers is superimposed and focused onto the semiconductor photomixer, inducing photocurrents oscillating at the difference frequency of the lasers, which is chosen in the terahertz range. An integrated antenna structure is employed to radiate the photocurrents as an electromagnetic free-space terahertz wave. Because of the antenna integration, such photomixers are commonly referred to as photoconductive antennas (PCAs) or photodiode antennas (PDAs). One of the prevalent CW photomixer technologies are InGaAs-based PiN-photodiodes exited by 1550 nm semiconductor lasers. Technical components for this so-called telecom wavelength, have benefited from intensive original research and development on fiber-optical communication, which in turn results in overall compactness, reliability, and cost efficiency of the respective terahertz systems^18^.

In fact, the emission spectra of CW Lasers show etalon effects inside the laser cavities yielding additional sidemodes besides the laser’s main mode at the targeted laser frequency. Depending on the level of suppression of such additional sidemodes, they may induce unintended mixing frequencies in a photomixer in addition to the desired terahertz output frequency defined by the difference frequency of the main modes of the superimposed lasers. These parasitic spectral components are in most cases especially pronounced on the very lower end of the terahertz frequency spectrum of the photomixer source, where the mixing efficiency is large. Combination of the source with a broadband power detector, such as a Golay cell, pyroelectric detector, TeraFET, Schottky diode, etc. with some sensitivity in this low-frequency regime can then contaminate measurement results at an intended frequency with an background signal due to the unwanted sidemode mixing effects. Therefore, for reliable detector calibration experiments or spectroscopic measurements, such parasitic frequency components must be considered. Their relative amplitude in the source’s power spectrum is of great importance and countermeasures to filter and/or suppress these unwanted source contributions should be taken. Especially in light of the typical roll-off to higher frequencies, the emitted power spectrum of sidemode mixing in the lower frequency range can be of a comparable order of magnitude as the higher frequency terahertz signal. We note here, that the intentional exploitation of multi-mode laser mixing in terahertz photomixers has been reported in the literature^7,22^ as well as the use of incoherent broadband light sources to drive the photomixers^23^.

In the literature on frequency-resolved characterization of terahertz detectors, the above effects are scarcely addressed. However, a close inspection of the measurement results reveals this underlying problem which authors may not be fully aware of at all times. In this contribution we show clear evidence for sidemode mixing in typical, commercially available PiN-PD photomixer sources for CW terahertz generation and present the associated challenges, when such devices are used for terahertz detector characterization. We also present a possible methodological approach to avoid the unwanted influence of parasitic mixing signals on broadband terahertz measurements.

## Results

**Figure 1:**
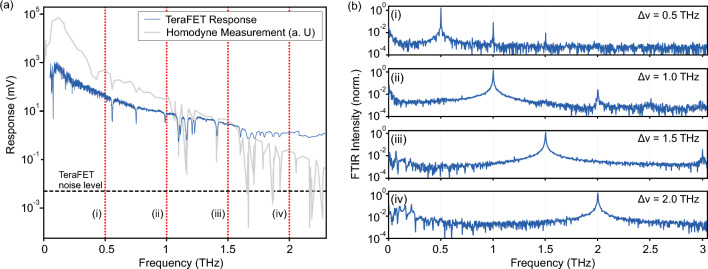
(**a**) TeraFET detector measurement with the setup illustrated in Fig. 5 and acquired with a lock-in amplifier. The dashed horizontal line marks the noise level of the detector. The red dashed vertical lines indicate the laser difference frequency settings, where additional FTIR spectra of the photomixer output were acquired. (**b**) Fourier-transformed FTIR measurements with the lasers configured for difference frequencies $\Delta \nu$ as given and labeled i–iv in (**a**). Normalized to main emission peak.

We observe in our investigations clear indications for sidemode mixing and higher harmonics in a typical terahertz photomixer as widely employed in terahertz CW spectroscopy systems. Figure 1a shows the direct detection response of a broadband Si-CMOS-based TeraFET detector (comparable to Ikamas et al.^12^) measured with the setup illustrated in Fig. 5. The CW terahertz radiation was generated with a InGaAs PiN-PD photomixer illuminated by two wavelengths-tunable CW lasers with operational wavelengths around 1550 nm, one being fixed (CoBrite DFB laser) in frequency and the second tuned (Finisar Y-Branch laser) to yield a resulting terahertz bandwidth up to 3 THz (details in the Methods section). This first measurement already reveals suspicious behavior as the detection signal follows the exponential decrease of the PDA output power only until approximately 1.25 THz and then transitions into a plateau towards higher frequencies. However the signal level remains well above the TeraFET’s noise level, which indicates a signal background due to parasitic frequency components in the source spectrum. For comparison, the light gray curve shows results of a coherent measurement of the same photomixer system with a PCA as homodyne detector fed by the same laser signal and confirming the expected overall exponential trend. Supporting the suspicion is the rather weak manifestation of water vapor absorption lines above 1 THz. An example of a proper representation of water vapor absorption in a broadband terahertz measurement can, e.g., be found in^18^. Note that the argument is not related to the absolute amount of water vapor and absolute depth of the lines, but rather the relative depth with respect to adjacent lines and the noise level of the TeraFET detector.

In order to further investigate this behavior of the terahertz photomixer, we performed spectrally resolved measurements of the photomixer emitter for fixed laser difference frequencies. The individual plots (i)–(iv) in Fig. 1b show emission spectra with the PDA tuned to 0.5, 1, 1.5, and 2 THz, respectively. The spectra were recorded with the help of a Michelson-type FTIR as illustrated in Fig. 4 (details described in the Methods section). In the experiment, a Golay cell was used as detector offering a nearly flat frequency response over the measured terahertz range^24^. Each measurement (i)–(iv) shows a clear peak correlating with the difference frequency $\Delta \nu$ of the main laser lines. However, we observe two additional, significant contributions to the overall output spectrum. First, a relatively broad spectral emission on the very low end of the spectrum beginning at around 50 GHz and extending, for measurement (iv), up to approximately 300 GHz. We will discuss in some detail below the source of these low-frequency output power contributions. Second, we clearly see pronounced peaks at higher harmonics of the target frequencies—most clearly in measurements (i), (ii) and (iii) at 1 THz, 2 THz and 3 THz, respectively. The higher harmonic of the measurement at 2 THz difference frequency (iv) lies well outside the covered measurement bandwidth. Note that the parasitic frequency contributions are only  15 dB suppressed compared to the output power levels at the configured target terahertz frequencies. With the high dynamic range of broadband TeraFET detectors such parasitic signals in the terahertz emitter’s output spectrum can lead to a significant offset on the detection signal and serve as an explanation for the missing water vapor lines and the signal plateau in Fig. 1a.

We now focus on the broad, low-frequency signals which seem to be mainly responsible for the signal offset we observe in *naive*, straight-forward experiments for the characterization of power detectors. Therefore, we inspected the optical output spectrum of both employed lasers—individually and combined—of the photomixer system with an optical spectrum analyser (OSA). The laser outputs are connected to the OSA by a 1550 nm single mode polarization maintaining optical fiber and an additional 10 dB power attenuator to work inside the operational power range of the OSA.

**Figure 2:**
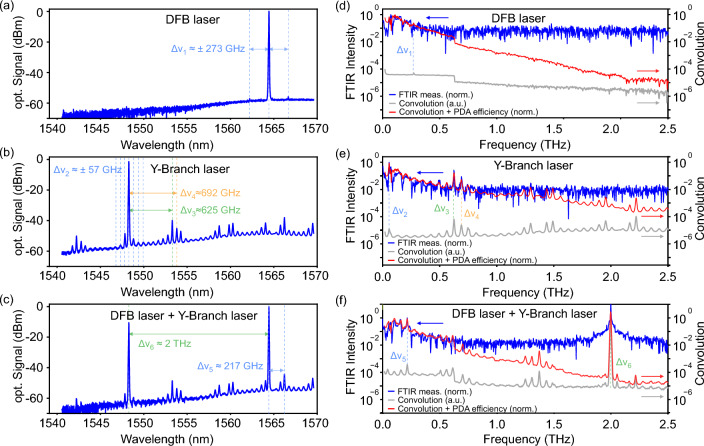
Left: Amplified laser spectra of the DFB (CoBrite) (**a**) and the Y-Branch Laser (Finisar) (**b**) lasers as well as the combined spectrum (**c**), measured with an OSA. The DFB laser is fixed at 1564 nm (191.6 THz). The spectrum of the Y-Branch Laser is displayed for a setting of 1548 nm (193.6 THz). Right: Measured FTIR output spectra (blue curves) of the photomixer when driven with the respective lasers, DFB laser (**d**), Y-Branch Laser (**e**), and combined (**e**), and normalized to low-frequency parasitic components. In each graph, the calculated autoconvolution spectrum of the laser signal is given as light gray curve (a.u.). The slope of the autoconvolution is corrected (red curve) with frequency-dependent terahertz output power of the photomixer. For all three measurements, we find excellent qualitative agreement between the measured and calculated spectra.

Figure 2 shows the acquired OSA laser power spectra of the CoBrite DFB laser (a), the Finisar Y-Branch laser (b), and both lasers combined with a fiber coupler (c), respectively. The spectra were measured after an erbium doped fiber amplifier (EDFA) which is used in the terahertz experiments to ensure sufficient optical power on the photomixer. We find that the DFB laser—at fixed wavelength of 1564.4 nm—offers good sidemode suppression of at least 56 dB of two recognizable sidemodes at 1562 nm and 1567 nm, respectively $\Delta \nu _1 \approx \pm \, 273$ GHz distance from the main mode. On the other hand, the spectrum of the Y-Branch laser shows relatively pronounced, equidistant sidemodes throughout the entire measured spectral range with a frequency spacing of approximately $\Delta \nu _2 \approx \pm \,57$ GHz—most noticeable around the main peak—as well as a number of bundled sets of larger sidemodes, indicated here, as an example, at $\Delta \nu _3 \approx \pm \, 625$ GHz and $\Delta \nu _4 \approx \pm \, 692$ GHz. Here, the minimum sidemode suppression is only around 47 dB. Finally, the combined laser spectrum, as it is used in the photomixer system, also exhibits significant sidemodes besides the two main laser lines. The spectrum is dominated by the characteristics of the Y-Branch laser but also contains additional difference frequency combinations, e.g., at $\Delta \nu _5 \approx 217$ GHz, between the DFB laser’s main lobe and the Y-Branch laser’s sidemodes. Note that in all three measurements, the overall signal decrease towards lower wavelength results from the frequency dependent gain of the EDFA. From the OSA measurements alone, it must already be suspected that additional mixing components besides the intended difference frequency $\Delta \nu _6 = 2$ THz of the main laser lines may be present in the terahertz output spectrum of a photomixer illuminated by these lasers.

We followed the above presumption in further detail by directly measuring the FTIR output spectrum of the photomixer when illuminated with the laser signals from Fig. 2a–c. Again, the setup illustrated in Fig. 4 was used, the results are shown in Fig. 2d–f. For an ideal, single CW laser with sufficient sideband suppression, no terahertz emission is expected from a photomixer. However, in measurements (d) and (e) where only a single laser is present, significant output of the terahertz emitter can be measured at the lower end of the frequency range around approximately 50 to 250 GHz. We attribute these signals to self-mixing of the lasers in the PiN-PDA of the photomixer, even for the supposedly good suppression of the DFB laser. The presence of self-mixing frequency components in the measurement with the Y-Branch laser is even more evident. The formation of a number of bundled emission peaks in accordance with the laser spectrum in Fig. 2b is strong evidence for the self-mixing assumption.

To further substantiate this, we calculate the autoconvolution of the photomixer’s input spectrum in order to estimate the frequency components from the non-linear photomixing process in the terahertz emitter. In a photomixer, the power of the generated terahertz radiation is proportional to the squared intensity $I^2_\text {opt}(t)$ of the optical excitation^7,22,25^:

1 $$\begin{aligned} P_\text {THz}(t)\propto I^2_\text {opt}(t) \propto |E_\text {opt}(t)|^4 \end{aligned}$$

where ${E}_\text {opt}(t) = E_{1}(t) + E_{2}(t)$ is the time domain electric field of the two superimposed lasers. In most theoretical treatments, the fields are assumed as ideal single-frequency laser lines $E_{1/2}(t) = E_{1/2}\cos \omega _{1/2} t$. However, from our OSA measurements, we deduce that the whole laser spectrum

2 $$\begin{aligned} E_\text {opt}(\omega ) = \sum _{i} E^i_1\cos \left( \omega _{i} t\right) + \sum _{i} E^i_2\cos \left( \omega _{i} t\right) \end{aligned}$$

with frequency components $\omega _i$ from both lasers should be considered. For each laser combination (a)–(c), we calculate the autoconvolution of the respective OSA spectrum to find theoretical self-mixing frequency components in the photomixers output spectrum (light gray curves in the figure). In order to account qualitatively for the overall frequency dependence of the terahertz output power of the photomixer, we correct the obtained curves with the slope of a terahertz power measurement of the same source (red curves).

For the DFB laser (Fig. 2d), the modelled curve reflects well the observed lower frequency components of the output spectrum up to roughly 250 GHz.The broad low frequency emission reflects the mixing between the main lobe of the DFB laser with its spectral noise floor. However, no significant signal peak can be recognized at the difference frequency of the lasers main and sidemodes at $\Delta \nu _1 = 273$ GHz, since the photomixing efficiency at this frequency is already to weak to be detected with the limited sensitivity of our FTIR. The situation becomes even more evident for the Y-Branch laser’s self-mixing spectrum shown in Fig. 2e. In particular, a signal is present at around $\Delta \nu _2 \approx \pm \, 57$ GHz, in accordance with the equidistant frequency spacing of the Y-Branch laser’s sidemodes. The characteristic bundles of sidemodes of the laser spectrum are also recognized in the modelled photomixer output spectrum and match well—shown as an example for $\Delta \nu _3$ and $\Delta \nu _4$—up to approximately 1.3 THz with the measured FTIR signal. Finally, Fig. 2f shows the output spectrum of the terahertz emitter in standard configuration with both lasers combined and set to a difference frequency of $\Delta \nu _6 = 2$ THz. Again, the measured terahertz spectrum is in good qualitative agreement with the modelled output frequency characteristics. For example, a signal peak at $\Delta \nu _{5}$ can be identified as the mixing signal of the DFB laser’s main mode and a prominent sidemode of the Y-Branch laser. Note that several other sidemode mixing frequency components are visible in the measured spectra which match well with the calculated curves, yet, we decided to mark only a few most pronounced ones to not overload the graphics.

## Discussion

We have identified parasitic frequency components besides the configured difference frequency of the excitation lasers in the output spectrum of a photomixer source for CW terahertz generation. We have laid out our findings from detailed investigation of the involved lasers and the photomixer output in both theoretical considerations and measurements with a terahertz FTIR spectrometer. There is strong evidence that the parasitic frequency components arise from self-mixing of sidemodes of—in our case, mainly one (Finisar Y-Branch laser)—of the two lasers as well as higher harmonic mixing signals. Regarding the higher harmonics, we assume that these components are solely resulting from the nonlinear response of the PDA, since we did not observe any signals in the laser spectra that could also contribute to the parasitic emission.

Note that the peak radiation levels of the parasitic frequency components observed in the measurements in Fig. 1b as well as Fig. 2f are only two to three orders of magnitude smaller than the intended output frequencies defined by the configured laser’s main lobe difference frequencies. This is well within the sensitivity range of typical direct terahertz detectors and can therefore easily lead to problems and misinterpretations in terahertz power measurements as discussed above. Such parasitic signals can mask spectral features in CW spectroscopy, as was shown at the example of water vapor absorption lines in Fig. 1. We emphasize that the knowledge of such effects is just as crucial when characterizing the broadband spectral response of direct terahertz detectors with CW photomixer-based terahertz sources. In other words, it must be verified in such situations whether an employed CW terahertz photomixer source can indeed be considered a *continuous wave*, i.e., single-frequency terahertz source at all.

Finally—and to once more support our previous claims—we present one possible, straightforward approach to reduce the influence of the lower frequency parasitic spectral components. For this purpose, we use again the setup shown in Fig. 5 adding an aperture filter in the intermediate focal plane of the two off-axis paraboloidal mirrors to act as a simple high-pass filter for the terahertz radiation. The aperture filters parasitic signal components at the lower end of the terahertz emission spectrum, with the cut-off depending on the aperture’s exact diameter.

**Figure 3:**
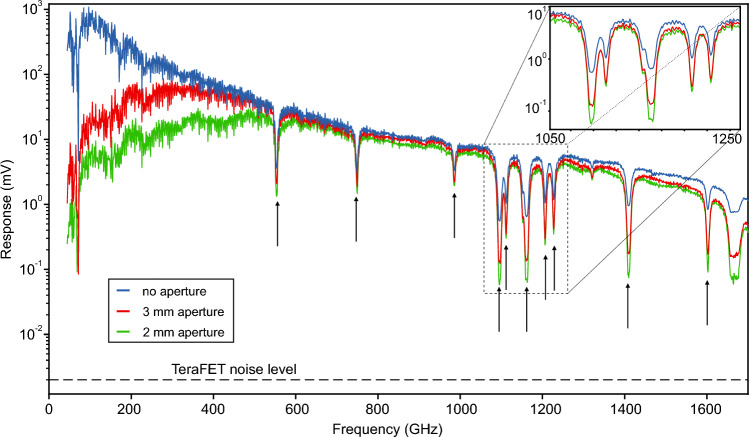
Broadband CW spectral measurements of the photomixer source with a TeraFET detector (blue). The measurement was repeated for different apertures sizes of 3 mm (red) and 2 mm (green) diameter in the intermediate focal plane. Black arrows mark the positions of water vapor absorption lines. The inset shows a zoom of the lines in the vicinity of 1.1 THz. The influence of the aperture filter on the depth of the absorption lines is clearly visible.

Figure 3 shows broadband CW measurements of the output power of the photomixer source acquired with a TeraFET detector and by sweeping the laser difference frequency over a range from 50 GHz to 1.75 THz. The measurements were performed in normal ambient air with three different aperture situations of 2 mm diameter, 3 mm diameter, and no aperture in the terahertz beam at all (the latter measurement in blue is the same as shown before in Fig. 1a). The characteristic water vapor absorption lines help to visualize the effect of parasitic frequency components of the photomixer source on CW spectroscopic measurements. Various absorption lines are clearly visible in all measured terahertz spectra indicated by the arrows. The figure inset shows a magnification of the absorption lines around 1.1 THz. Upon closer investigation, it is revealed that the depths of the absorption lines are strongly reduced in the unfiltered measurement without aperture (blue curve) due to the parasitic signal background discussed above. Adding the aperture in the intermediate focus has two effects: (i) the measurement signal is decreased in the lower frequency region, confirming the high pass character of the aperture filters, and (ii) the absorption lines of the water vapor become increasingly pronounced for smaller aperture sizes—approximately by 10 dB for an aperture of 2 mm diameter. The latter effect is most evident for the lines above 1.6 THz where the onset of a signal plateau in the unfiltered (blue) measurement can be recognized (compare again Fig. 1a) indicating that here, the measurement signal at the respective laser difference frequency and the parasitic low-frequency signals have equal amplitude. We note that measurements with a comparable setup (but different lasers) were performed in the past where similar observations were made^26^. It is important to emphasize, that an aperture filter only rejects the parasitic lower frequency components, while higher harmonics might still be present in the spectrum, which could still impact on the correct measurement of spectral absorption lines. These effects were not observed in our CW-Measurements, which might be explained by the relatively low signal power in these modes, in comparison to the signal power in the broad low frequency parasitic components.

### Conclusion

To conclude, we have demonstrated in a number of measurements clear evidence for the presence of parasitic frequency components in the output spectrum of typical CW terahertz photomixer sources. In our experiments, we were able to present strong indications for the origin of such signals, namely the self-mixing of laser sidemodes besides the main mixing component of the difference frequency of the two laser main modes. Our work therefore brings attention to an often overlooked issue when using such photomixer terahertz sources for power detection experiments. In particular, when broadband terahertz detectors such as golay cells, TeraFETs, pyroelectric detectors, etc. are used, the parasitic frequency components in the emitter’s output spectrum can significantly distort the measurement results. This can lead to far-reaching misinterpretations of measurement results. We showed that unwanted output frequencies are most pronounced in the lower frequency range < 0.25 THz where the photomixer’s efficiency is large and in many cases the employed detectors are especially sensitive. At the example of weak water vapor absorption lines, we could emphasize the importance of awareness of such unwanted signal contributions, which can mask characteristic spectral features in spectroscopic analyses. In particular at higher frequency settings of a photomixer system—in our case, approximately at laser difference frequencies larger than 1.5 THz—the parasitic frequency contributions in the spectrum can be as high in magnitude as the main signal, which manifests in the onset of a signal plateau. In theory, the effect of the higher harmonics in the CW spectra can lead to similar effects, such as the shallowing of absorption dips and the generation of fake absorption lines. In our measurements, we did not observe such effects, most likely due to the relatively low power at these harmonic frequencies, in contrast to the broad low frequency parasitic components.

We presented one simple but nevertheless useful approach to overcome some of the discussed problems. Introducing an aperture filter to cut low frequency signal components can help to increase the validity of measurement results at higher frequencies by removing signal offsets due to parasitic spectral contributions of the photomixer source. In our demonstration, we used a simple aperture in an intermediate focus of the free-space terahertz beam. In another project, we are currently working towards a permanent implementation of high pass aperture filters in TeraFET detectors by using a laser writing process to write metallic pinhole apertures directly onto the chips^27^. Another approach could be the implementation of filters already in the photomixer and/or detector designs.

Note once more that the observations in this work are only made in direct power detection but not present in heterodyne system concepts, where also, a superior NEP can be achieved^28^. If possible, the detector should be driven in a heterodyne measurement mode, avoiding the simultaneous detection of useful signal and parasitic frequency components. Although the influence of sidemode mixing signals in terahertz photomixer sources is discussed in this paper on the example of a TeraFET detector, the same effects should also be noticeable with other types of power detectors and eventually have to be considered in homodyne measurement schemes. It is therefore generally advisable when using terahertz photomixers to investigate the influence of possible sidemode mixing for the applications addressed.

## Methods

### PiN-PD Photomixer

In common CW terahertz photomixer emitters, two wavelength-tunable single-mode lasers with frequencies $\nu _{1}$ and $\nu _{2}$ are superimposed, resulting in a fast carrier frequency and a slow beat or difference frequency ($\nu _\text {diff}=|\nu _{1}-\nu _{2}|$). The combined laser signal is focused onto a suitable semiconductor structure inducing an alternating photocurrent at the difference frequency of the lasers. Integrated planar antenna structures are used to radiate the generated electric field into free space. Tuning of the difference-frequency $\nu _\text {diff}$ of the lasers translates directly into the tunability of the terahertz radiation of these sources. The wavelengths of the laser excitation determines appropriate material systems with respect to band-gap properties of the semiconductor. For instance, photomixers based on Indium Gallium Arsenide (InGaAs) are commonly used with 1550 nm laser systems, while Gallium Arsenide (GaAs) based transmitters usually work with 850 nm radiation. Especially the use of 1550 nm optical lasers allows to benefit from the maturity of technological components for this wavelength. Components, which were originally developed for fiber-optical communication, can find their use in 1550 nm terahertz CW systems, which results in overall compactness, reliability, and cost efficiency^18,20^. The so called PiN photodiode (PiN-PD) is one of the commonly used types of CW emitters for the 1550 nm excitation wavelength. The structure of a PiN-PD is composed of an intrinsic absorption layer sandwiched between n-type and a p-type semiconducting layers. Photocarriers are exited in the intrinsic layer by laser illumination and accelerated by the intrinsic electrical field and an additional external bias field forming an alternating photocurrent, which can be irradiated by an appropriate antenna structure. For our experiments, we use an InGaAs waveguide-integrated PiN-PD (WiN-PD) as photomixer source for the generation of terahertz radiation, similar to the one in^29,30^. The transmitter is driven by two tunable 1550 nm semiconductor lasers.

### Laser sources and Amplifier

Two tunable 1550 nm lasers are part of the CW terahertz photomixer system we used for the investigations in this paper. The first laser is a CoBrite CBDX1-1-C-H01-FA narrow linewidth ($\approx$ 80 kHz), wavelength tunable CW laser with a wavelength range from 1527.60 nm to 1568.6 nm. For the broadband terahertz spectral measurements, this laser is kept at fixed wavelength of 1564.4 nm (191.63 THz). The second employed laser is a Finisar “Modulated Grating Y-Branch Laser”, where the laser cavity is split into two paths (by a Y-junction) with parallel Bragg reflectors on each end. The reflectors are modulated gratings with characteristic multi-peak reflection spectra. Both reflections are combined in a multimode interference coupler at the Y-junction, leading to the additive vernier effect^31^. This laser concept offers broad spectral coverage and fast wavelength tunability. In our measurements presented in this work, we tuned the laser’s wavelength from 1564.0 to 1540.3 nm (191.68 to 194.63 THz) to cover a bandwidth from 50 GHz to 3 THz. In principle, a maximum terahertz tuning range of approximately 5 THz can be covered with this laser combination. For amplification of the optical signals, we use an erbium-doped fiber amplifier (EDFA).

### Terahertz FTIR setup

**Figure 4:**
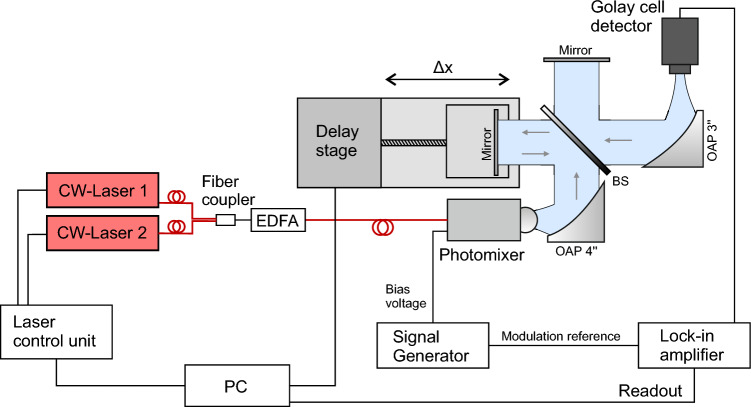
FTIR measurement setup for spectral measurements of the CW photomixer output. The two lasers are combined in a fiber coupler, amplified by an EDFA, and then fed to the photomixer. A Golay cell is used to record the terahertz signal from the Michelson-type interferometer with PC-controlled mechanical delay line. The photomixer’s bias voltage is modulated at 20 Hz for lock-in readout of the detection signal.

To investigate the output spectrum of the terahertz photomixer source, we performed FTIR measurements as presented in Figs. 1b and 2d–f. Figure 4 illustrates the employed setup of a Michelson interferometer for the FTIR measurements. For each measurement, the two lasers are set to a fixed difference frequency with a laser control unit, superimposed in a fiber coupler, and amplified to approximately 30 mW optical power with an EDFA, and fed to the PiN-PD photomixer. An Off-axis paraboloidal mirror (OAP) is used for the collimation of the terahertz radiation emitted by the antenna. The collimated terahertz beam is then divided by a high resistive silicon wafer with a thickness of 280 ${\upmu }$m into two separate beam paths, one with constant path length using a fixed flat mirror, the other one serving as a variable delay line with another flat mirror mounted on a PC-controlled linear translation stage. Our stage provided a maximum path difference of 50 mm at 0.1 ${\upmu }$m minimum resolution, yielding a total bandwidth of 1499 THz with a resolution of approximately 3 GHz in the FTIR measurements. In all our FTIR measurements, we use a step width of 0.02 mm and 50 mm total displacement yielding a maximum frequency measurement bandwidth of around 3.7 THz. The collimated terahertz beams from both paths are spatially superimposed by the silicon beam splitter (BS) after reflection from the planar mirrors. A second OAP with focal length of 3” is then used to focus the terahertz beam onto the detector input aperture. For the measurements presented in this work, we used a Golay cell detector with assumed flat broadband responsivity over the relevant frequency range. We modulate the bias voltage of the photomixer source with the help of a signal generator at 20 Hz and use a lock-in amplifier to record the interferometric terahertz signal with 1 s integration time at each position of the translation stage.

To investigate the influence of single-laser sidemode mixing, we also performed the FTIR measurements using only one of the lasers at a time to drive the photomixer source. To ensure that the optical power is constant in all measurements, we used the EDFA to adjust the power to an equal level of total laser power on the photodiode. For the direct investigation of the lasers’ spectra shown in Fig. 2a–c we measured the output of the EDFA with an OSA.

### Terahertz direct detection measurements

**Figure 5:**
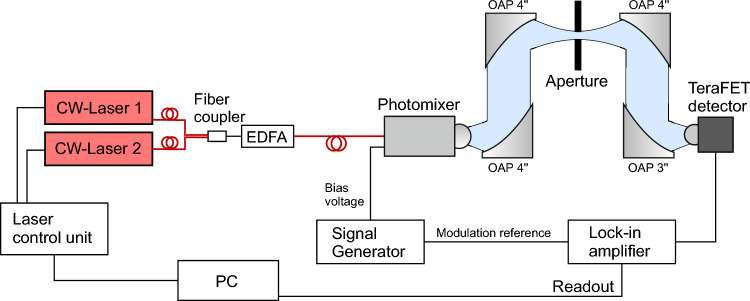
Terahertz CW measurement setup with a TeraFET as direct power detector. Terahertz generation in the photomixer is similar as in the FTIR setup above. Again, a lock-in is used for signal readout, here, at 1.333 kHz modulation frequency. An aperture is introduced as high pass filter in the intermediate focus of the terahertz beam to filter parasitic signal components at lower frequencies.

Figure 5 illustrates a typical CW terahertz photomixer system setup as it is widely used for spectrally resolved terahertz measurements in scientific and industrial applications. We used a similar setup for our measurements shown in Figs. 1a and 3. Laser control and generation of the CW terahertz radiation follows the same principle as in the FTIR setup discussed above. Here, we use four OAP mirrors to generate an intermediate focus in the terahertz beam. For the measurements shown in Fig. 3, we placed an additional aperture in the intermediate focal plane for filtering out lower frequency parasitic signal components. An OAP with 3” focal length is used to focuses the terahertz beam onto the detector aperture for optimum coupling into the employed TeraFET detector with hyper-hemispherical substrate lens^13,24^. Spectral measurements are performed by sweeping the difference frequency of both lasers driving the photomixing antenna, where one laser (Cobrite DFB laser) is kept at a fixed wavelength (1564.4 nm) and the second laser (Finisar Y-Branch laser) is tuned over a 3 THz range with respect to the first laser. Measurements with the setup were performed with 100 ms lock-in integration time at 1.333 kHz square wave modulation of the photomixer’s bias voltage.

## Data Availability

The datasets generated during and/or analysed during the current study are available from the corresponding author on request.
